# Dietary Fiber Hierarchical Specificity: the Missing Link for Predictable and Strong Shifts in Gut Bacterial Communities

**DOI:** 10.1128/mBio.01028-21

**Published:** 2021-06-29

**Authors:** Thaisa M. Cantu-Jungles, Nuseybe Bulut, Eponine Chambry, Andrea Ruthes, Marcello Iacomini, Ali Keshavarzian, Timothy A. Johnson, Bruce R. Hamaker

**Affiliations:** a Whistler Center for Carbohydrate Research and Department of Food Science, Purdue University, West Lafayette, Indiana, USA; b Department of Biochemistry and Molecular Biology, Universidade Federal do Paraná, Curitiba, Brazil; c Department of Internal Medicine, Division of Gastroenterology, Rush University Medical Center, Chicago, Illinois, USA; d Department of Animal Sciences, Purdue University, West Lafayette, Indiana, USA; Corporación CorpoGen

**Keywords:** dietary fiber, fermentation response, gut microbiota, microbial community, prebiotic, short-chain fatty acids

## Abstract

Most dietary fibers used to shape the gut microbiota present different and unpredictable responses, presumably due to the diverse microbial communities of people. Recently, we proposed that fibers can be classified in a hierarchical way where fibers of high specificity (i.e., structurally complex and utilized by a narrow group of gut bacteria) could have more similar interindividual responses than those of low specificity (i.e., structurally simple and utilized by many gut bacteria). To test this hypothesis, we evaluated microbiota fermentation of fibers tentatively classified as low (fructooligosaccharides), low-to-intermediate (type 2 resistant starch), intermediate (pectin), and high (insoluble β-1,3-glucan) specificity, utilizing fecal inoculum from distinct subjects, regarding interindividual similarity/dissimilarity in fiber responses. Individual shifts in target bacteria (as determined by linear discriminant analysis) confirmed that divergent fiber responses occur when utilizing both of the low-specificity dietary fibers, but fibers of intermediate and high specificity lead to more similar responses across subjects in support of targeted bacteria. The high-specificity insoluble β-glucan promoted a large increase of the target bacteria (from 0.3 to 16.5% average for *Anaerostipes* sp. and 2.5 to 17.9% average for Bacteroides uniformis), which were associated with increases in ratios of related metabolites (butyrate and propionate, respectively) in every microbial community in which these bacteria were present. Also, high-specificity dietary fibers promoted more dramatic changes in microbial community structure than low-specificity ones relative to the initial microbial communities.

## INTRODUCTION

Diet is known to be an important factor able to shape gut microbial communities to potentially promote colonic and systemic health (1). Fermentable dietary fibers, which are not digested in the upper gastrointestinal tract, are important energy and carbon sources to commensal bacteria residing in the colon. We postulated that discrete chemical and physical structures of fibers can act to align bacteria to fibers and govern their ability to utilize a given fiber (2). Such characteristics were recently proven to be an important factor to bring about direct and consistent fiber responses in humans (3). However, divergent interindividual responses to the same fiber supplement are often reported depending on the baseline gut microbial community composition (4). At the population level, it has not been clear what determines whether individuals’ microbiota respond differently or the same to fermentable dietary fibers.

Variability in response is not only related to the presence or absence of a given target bacterium in the community but also related to divergent background microbial communities affected by factors such as diet and host genetics (4). The overall gut microbiota composition establishes the competitive environment of the target bacteria for nutrient acquisition and utilization and was shown to be an important driving force to interindividual variability regarding fiber responses (5–7). Overlapping abilities of different microbes to ferment the same fiber structure, together with limited substrate availability relative to high gut bacterial density (5), make it hard to ensure that a given fiber will be utilized to promote the growth of a target microbe even if it has all the capabilities to utilize it. As an example, Patnode et al. (6) colonized gnotobiotic mice with 15 strains of bacteria from the human gut including the two arabinoxylan utilizers Bacteroides cellulosilyticus and Bacteroides ovatus. When mice were fed diets containing arabinoxylan, relative abundance of *B. ovatus* was increased only when its arabinoxylan competitor (B. cellulosilyticus) was omitted from the inoculated community, illustrating how competitive pressures can impair predictable bacterial responses to fiber.

Recently, we proposed a new hierarchical dietary fiber classification, and we hypothesized that some dietary fiber structures, which we classified as “high-specificity fibers,” can be selected to limit the number of gut bacteria capable of utilizing them and, hence, alleviate gut competitive pressures for the substrate (8). The reduced competition for high-specificity fibers would thereby promote increases in target bacteria independent of the overall background microbial community, allowing similar microbial shifts in subjects with distinctly different microbiota communities. Contrastingly, “low-specificity fibers,” which can be utilized by many gut microbes, generally would produce more individualized outcomes, resulting in enrichment of varied target bacteria depending on an individual’s microbial background community.

Factors that increase a fiber’s specificity and thus limit the number of bacteria able to utilize it were proposed to be high chemical and/or physical complexity, reduced commonality in diets, and fibers that are utilized through a limited network of cross-feeders (8). These factors may be combined and have distinct levels of importance in making a dietary fiber more or less specific. For example, physical complexity can be more important than chemical complexity, since it can limit bacterial access to the chemical structure that needs to be fermented. As such, even a chemically simple dietary fiber might be very specific if present in an insoluble form which limits the accessibility of the majority of bacteria able to degrade it. On the other hand, fiber physical complexity could become less important when intensively utilized through cross-feeding, where multiple bacteria benefit from the degradation of a fiber. An example is resistant starch, in the case of which a few primary degraders directly access and degrade it, but they support a number of secondary degraders (9), thus reducing the fiber’s specificity. In this proof-of-concept work, we used our hypothetical classification of dietary fibers with a combination of known features that can increase/decrease specificity to show that fiber specificity is in fact related to homogeneity of fiber response across distinct human gut microbial communities and also relates to the intensity of observed microbial shifts.

## RESULTS

### Baseline microbial composition and diversity across donors.

**FIG 1:**
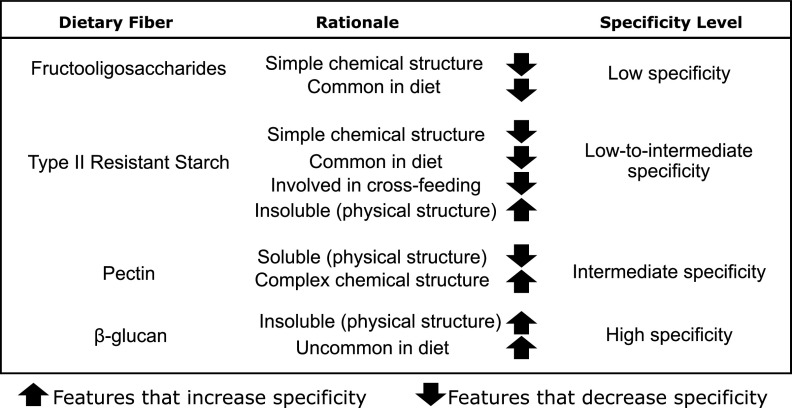
Rationale for the classification and selection of dietary fibers utilized for *in vitro* fecal fermentation in D1 to D10.

**FIG 2:**
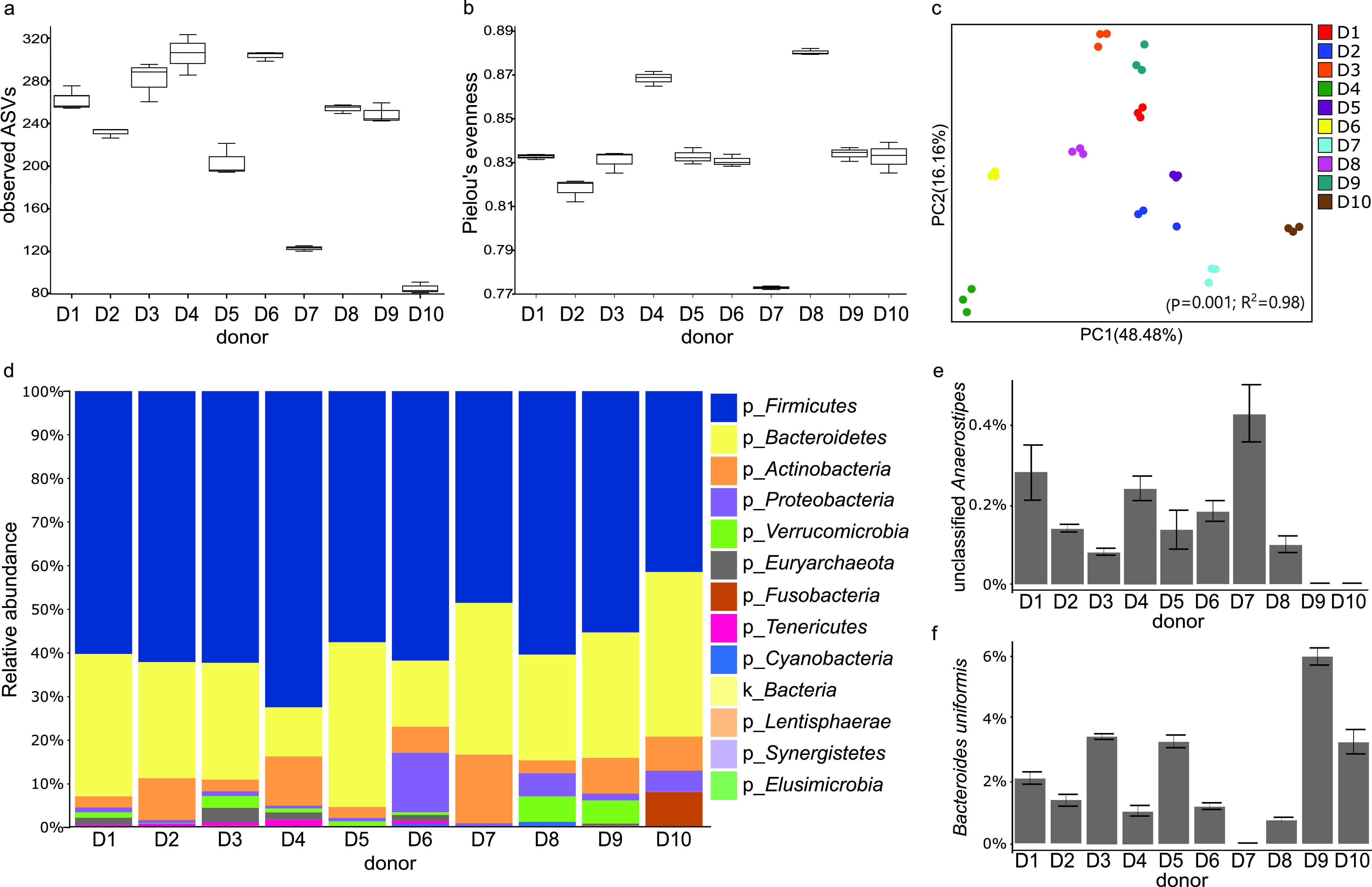
Baseline gut microbial communities from donors D1 to D10. (a) Number of observed ASVs. (b) Pielou’s measure of species evenness. (c) PCoA plot of weighted UniFrac measure of beta diversity. (d) Relative taxon abundance (%) at the phylum level per donor. (e) Relative taxon abundance (%) at species level of unclassified *Anaerostipes* relative per donor. (f) Bacteroides uniformis relative abundance (%) per donor.

Fecal samples were obtained from 10 healthy subjects (here referred to as “donors” [D]) to represent distinctly different gut microbial communities in which fermentation of fibers of lower or higher specificity was further performed. The rationale for selection and categorization of dietary fiber specificity used in this study is summarized in Fig. 1. High variability in the donor’s baseline alpha diversity was observed regarding both richness as determined by the number of amplicon sequence variants (ASVs) (*P* = 0.001, Fig. 2a), and evenness as determined by Pielou’s measure of species evenness (*P* = 0.005) (Fig. 2b). Permutational multivariate analysis of variance (PERMANOVA) using weighted and unweighted UniFrac beta diversity metrics indicated significant community dissimilarities among donors (*P* = 0.001). Variations among donors could be observed in both principal-coordinate analysis (PCoA) axes and were best explained by the weighted UniFrac PCoA (*R*^2^ = 0.98, Fig. 2c), suggesting a major effect of bacterial abundance on donor differentiation rather than by absence/presence (i.e., prevalence) of taxa alone as detected by the unweighted UniFrac PCoA (*R*^2^ = 0.93; see Fig. S2 in the supplemental material). Interindividual variability was also reflected in variations in taxon relative abundance, with *Firmicutes* ranging from 50% to 75%, *Bacteroidetes* from 11% to 32%, *Actinobacteria* from 2% to 13%, and *Proteobacteria* from 0.4% to 10%, depending on the donor (Fig. 2d). Overall, donors’ baseline microbial communities further utilized for fiber fermentation experiments (D1 to D10) presented high variability in community structure and composition.

10.1128/mBio.01028-21.2 FIG S2 Unweighted UniFrac plot of microbial beta diversity among donors (D1 to D10) at baseline. Download FIG S2, EPS file, 0.1 MB. Copyright © 2021 Cantu-Jungles et al. 2021 Cantu-Jungles et al. https://creativecommons.org/licenses/by/4.0/ This content is distributed under the terms of the Creative Commons Attribution 4.0 International license.

Since the high-specificity β-glucan fiber utilized in this study was previously demonstrated to have a targeted action toward an unclassified *Anaerostipes* species and Bacteroides uniformis (10), D1 to D10 were also evaluated regarding relative abundance of these species in a taxonomy-based analysis with ASVs collapsed at the species level (Fig. 2e and f). Unclassified *Anaerostipes* was present in very low abundance in D1 to D8 (from 0.08 to 0.45%), while no sequences were detected in D9 or in D10 (Fig. 2e). B. uniformis baseline abundance ranged from 0.7 to 5.7% with sequences detected in all donors, except for D7 (Fig. 2f).

### Fibers of higher specificity lead to similar bacterial shifts across donors.

**FIG 3:**
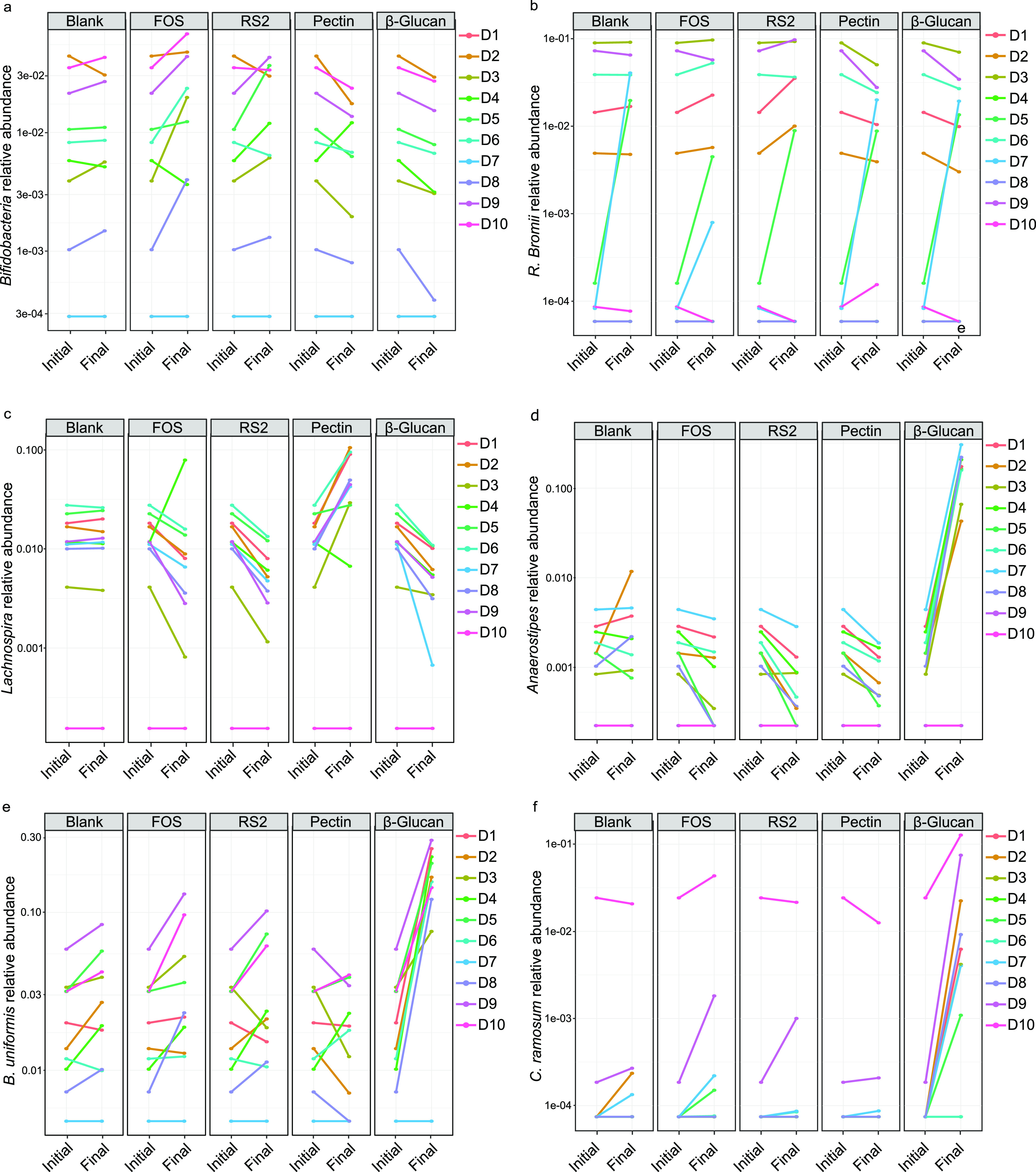
Relative abundance (where 1 = 100%) of taxa collapsed at the species level, before and after fiber fermentation in each donor, of unclassified *Bifidobacteria* (a), *R. bromii* (b), unclassified *Lachnospira* (c), unclassified *Anaerostipes* (d), B. uniformis (e), and *C. ramosum* (f). Note the *y* axes in all panels are on a log scale.

Fibers of low specificity (fructooligosaccharide [FOS]), low-to-intermediate specificity (type 2 resistant starch [RS2]), intermediate specificity (pectin), and high specificity (insoluble β-glucan) (10) were selected according to the rationale described in the methodology and summarized in Fig. 1 and submitted to 24-h *in vitro* fecal fermentations of D1 to D10. To test our hypothesis that gut fermentation of higher-specificity fibers leads to similar results in different microbial communities, while lower-specificity fibers lead to divergent outcomes, we evaluated consistency in responses of important taxa across donors. Bacterial species uniquely enriched in each fiber fermentation compared to the blank were identified through Analysis of Composition of Microbiomes-II (ANCOM-II) (Fig. S3), and the relative abundances of identified species were further evaluated in each individual donor before and after fiber fermentation (Fig. 3). No discriminant features were found for the low- and low-to-intermediate-specificity fibers (FOS and RS) tested in this study, confirming a lack of specificity of these fibers to specific bacteria when given to different donors. Analysis of responses of bacteria commonly associated with these fiber treatments such as *Bifidobacteria* for FOS (11) and Ruminococcus bromii for RS2 (12) showed different responses across donors, in accordance with the high interindividual variability expected for fibers of lower specificity (Fig. 3a and b).

10.1128/mBio.01028-21.3 FIG S3 Bacterial species identified as discriminant features in ANCOM performed in each fiber substrate versus the blank (no fiber added), using donors as random effect and adjusted for time (before versus after 24-h fermentation). Only taxa with a W statistic higher than 10 are presented in this figure. Download FIG S3, EPS file, 0.1 MB. Copyright © 2021 Cantu-Jungles et al. 2021 Cantu-Jungles et al. https://creativecommons.org/licenses/by/4.0/ This content is distributed under the terms of the Creative Commons Attribution 4.0 International license.

For the intermediate-specificity fiber (pectin), unclassified *Lachnospira* was identified through ANCOM-II as abundant compared to the blank (Fig. S3). Unclassified *Lachnospira* markedly increased after treatment with pectin in all donors, except for D4 and D10 (Fig. 3c). Because no unclassified *Lachnospira* sequences were detected in D10 at baseline (Fig. 3c), D4 was considered the only true nonresponder for the intermediate-specificity fiber.

For the high-specificity fiber (β-glucan), unclassified *Anaerostipes*, B. uniformis, and Clostridium ramosum were detected as significantly enriched in comparison to the blank (Fig. 3a) through ANCOM analysis (Fig. 3a). Compared with previous data from our group (data not shown), the same unclassified *Anaerostipes and* B. uniformis were found to be promoted by β-glucan fiber ferments using a pooled fecal sample (10). In the present study, individual analysis showed that these taxa were enriched in all donors who had detectable sequences of these bacteria before fermentation in a similar intensity (Fig. 3d to f). Unclassified *Anaerostipes* substantially increased from only 0.2% average relative abundance before fermentation to 15.5% average relative abundance after 24-h fermentation with the β-glucan fermentation (Fig. S4). Only D9 and D10 did not present unclassified *Anaerostipes* growth after β-glucan fermentation due to their lack of these bacteria at baseline (Fig. 3d). Also, enrichment of unclassified *Anaerostipes* was not observed in any of the donors treated with the fibers of lower specificity. Likewise, B. uniformis was enriched in a similar intensity in donors after 24-h fermentation (from 2.4 to 17.9% average) and had no response in D7, who did not have any detectable B. uniformis sequences at baseline (Fig. 3e and Fig. S4). Although some donors presented increases in B. uniformis relative abundance with fermentation of other substrates, none were of a magnitude similar to that of the high-specificity β-glucan fiber. Clostridium ramosum was also similarly promoted in all donors fermented with the β-glucan (Fig. 3f), but this bacterial species represented a minor taxon in individuals harboring unclassified *Anaerostipes* (D1 to D8) and was more abundant only in those who did not have unclassified *Anaerostipes* (D9 and D10) (Fig. S4). The finding that unclassified *Anaerostipes* was enriched in the donor with no detectable sequences of B. uniformis (D7) and B. uniformis was enriched in donors with no detectable sequences of unclassified *Anaerostipes* (D9 and D10) (Fig. 3d and e) is indicative that these two bacteria did not rely on cross-feeding reactions between them to grow on the high-specificity fiber.

10.1128/mBio.01028-21.4 FIG S4 Relative abundances (%) at species level, for taxa identified as significant by differential analysis through ANCOM-II for the β-glucan, before and after fiber fermentations in each donor. Download FIG S4, EPS file, 0.1 MB. Copyright © 2021 Cantu-Jungles et al. 2021 Cantu-Jungles et al. https://creativecommons.org/licenses/by/4.0/ This content is distributed under the terms of the Creative Commons Attribution 4.0 International license.

**FIG 4:**
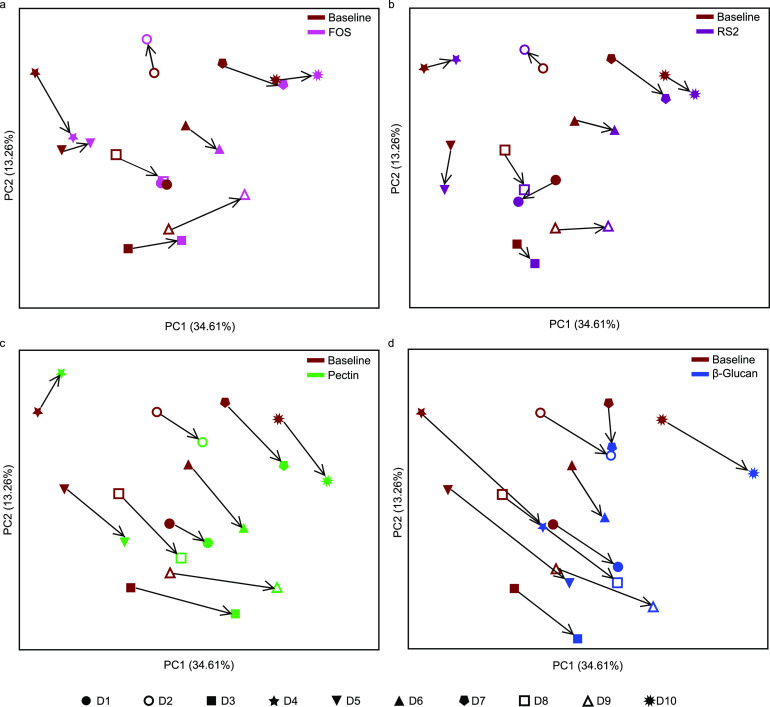
Weighted UniFrac beta diversity among donors in the baseline and all fiber ferments visualized separately in baseline and fructooligosaccharides (FOS) (a), baseline and type 2 resistant starch (RS2) (b), baseline and pectin (c), and baseline and β-glucan (d). Direction of change between baseline and fiber ferments is indicated with an arrow. Each dot represents a mean from biological triplicates.

To evaluate if similar responses in key bacteria also were reflected in the overall microbial community structures, we compared beta diversity metrics using weighted UniFrac distance matrix (Fig. 4 and Fig. S5). As hypothesized, shifts in beta diversity from baseline to 24-h fermentation of low- and low-to-intermediate-specificity fibers (FOS and RS2, respectively) occurred in different directions across individuals (Fig. 4a and b), and no clustering tendency was observed in comparison to the blank (Fig. S5a and b). For the intermediate-specificity fiber (pectin), however, there was a trend in donors’ shifts to the same direction (Fig. 4), leading to some clustering in the PCoA plot (Fig. S5c). For the high-specificity fiber (β-glucan), all donors moved in the same direction (toward the right bottom side of the plot) (Fig. 4d), presenting a clearer tendency of interdonor clustering at 24 h (Fig. S5d). Notably, for the β-glucan, D10 was the only outlier on the PCoA plot (Fig. S5d) and corresponds to a donor lacking in one of the target bacteria (unclassified *Anaerostipes*) (Fig. 2e). When we restricted our analysis to the seven donors harboring both *Anaerostipes* and B. uniformis, a significantly lower within‐donor dispersion was observed for the high-specificity β-glucan compared to that of the blank (permutational multivariate analysis of variance [PERMDISP] *q* = 0.003), whereas the lower-specificity fibers (FOS and RS2) presented the same dispersion across donors as the blank. Pairwise PERMDISP did not show significance between pectin and the blank (*q* = 0.26), likely due to the high sensitivity of the analysis to outliers (i.e., D4) (13). Thus, high-specificity fibers had a similarity in donor response and trended toward a convergence of the overall microbial structure among donors.

10.1128/mBio.01028-21.5 FIG S5 Weighted UniFrac plots of microbial beta diversity among donors in all substrates and the blank visualized separately in fructooligosaccharides (FOS) and blank (a), type 2 resistant starch (RS2) and blank (b), pectin and blank (c), and β-glucan and the blank (d). Each dot represents a biological replicate. Download FIG S5, EPS file, 0.1 MB. Copyright © 2021 Cantu-Jungles et al. 2021 Cantu-Jungles et al. https://creativecommons.org/licenses/by/4.0/ This content is distributed under the terms of the Creative Commons Attribution 4.0 International license.

Taken together, these data provide support for our hypothesis, as stated in the work of Cantu-Jungles and Hamaker (8), and showed that high-specificity fibers, but not lower-specificity ones, promote a similar fiber response independent of the background microbial community as long as the target bacteria are present.

### High-specificity fibers promote larger shifts in community structure than do low-specificity fibers.

**FIG 5:**
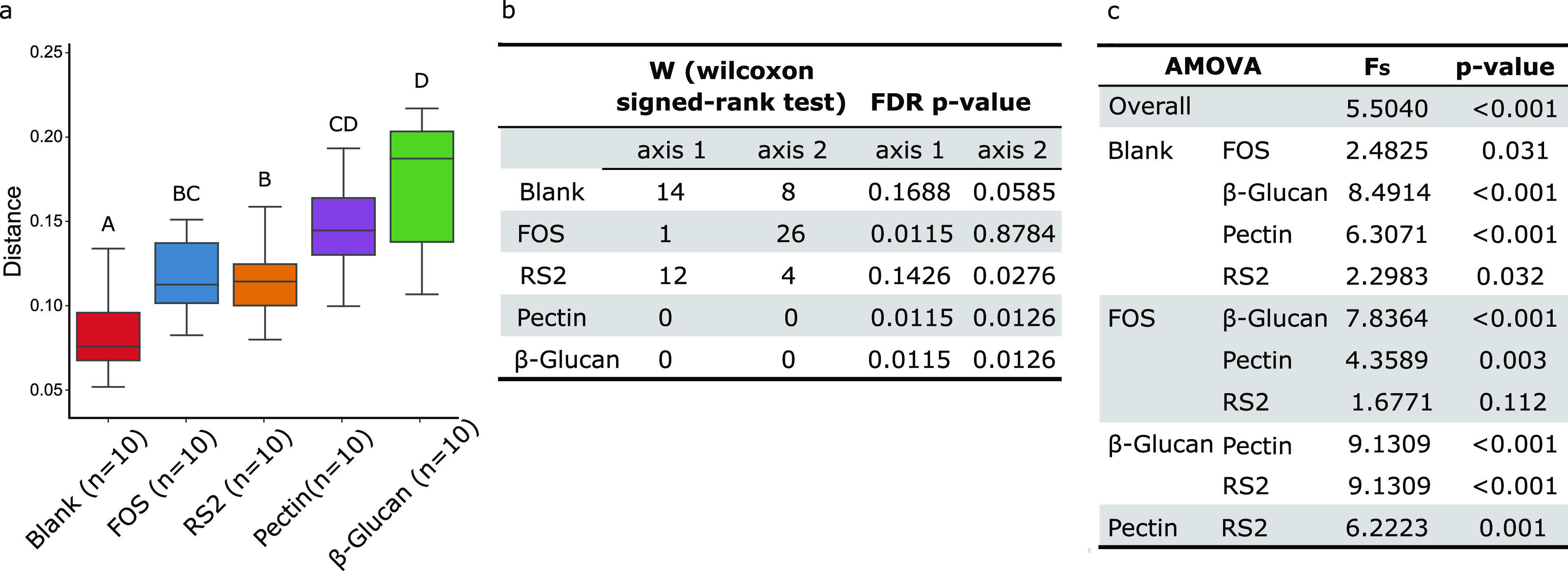
(a) Pairwise distances of the weighted UniFrac beta diversity metrics as calculated by the q2-longitudinal plugin. The fiber group is represented on the *x* axis, and pairwise distances between pre- and postfiber fermentations are demonstrated on the *y* axis. Different letters denote statistical significance (*P* < 0.05). (b) Significance of differences before and after fermentations in coordinates of the weighted UniFrac beta diversity PCoA calculated through q2-*longitudinal* plugin. (c) AMOVA pairwise comparison of weighted UniFrac distance matrix among substrates.

To evaluate if fibers of low, intermediate, and high specificity were different in their capacity to change overall community structure in comparison to its baseline, we performed a longitudinal and paired-sample analysis using weighted UniFrac beta diversity for each substrate through the QIIME2 longitudinal plugin. Distances from the baseline microbial community were higher for the high (β-glucan)- and intermediate (pectin)-specificity fibers than low-to-intermediate- and low-specificity ones (FOS and RS2) (Fig. 5a). Significance of differences in the PCoA coordinates before and after each fiber fermentation is presented in Fig. 5b. No significance in change of community structure over the 24-h fermentation was found for the blank, and fibers of lower specificity (FOS and RS2) presented significant differences (false-discovery rate [FDR] *P* value < 0.05) in only one of the two PCoA axes. Higher-specificity pectin and β-glucan fibers, on the other hand, showed significant differences in both axes.

Finally, analysis of molecular variance (AMOVA) was conducted using the distance matrix from weighted UniFrac beta diversity to evaluate how centroids’ location (taken alone) of the final microbial communities differed from that of the blank (Fig. 5c). Only fibers of higher specificity (pectin and β-glucan) presented significant changes in microbial structure with *P*_AMOVA_ values of <0.001. For lower-specificity fibers, RS2 and FOS presented significant differences only if a *P*_AMOVA_ value of <0.05 was considered. Thus, fibers of higher specificity promoted higher intensity of shifts in microbial communities than did those of lower specificity.

### Promotion of specific bacteria is associated with larger and similar SCFA shifts across donors.

**FIG 6:**
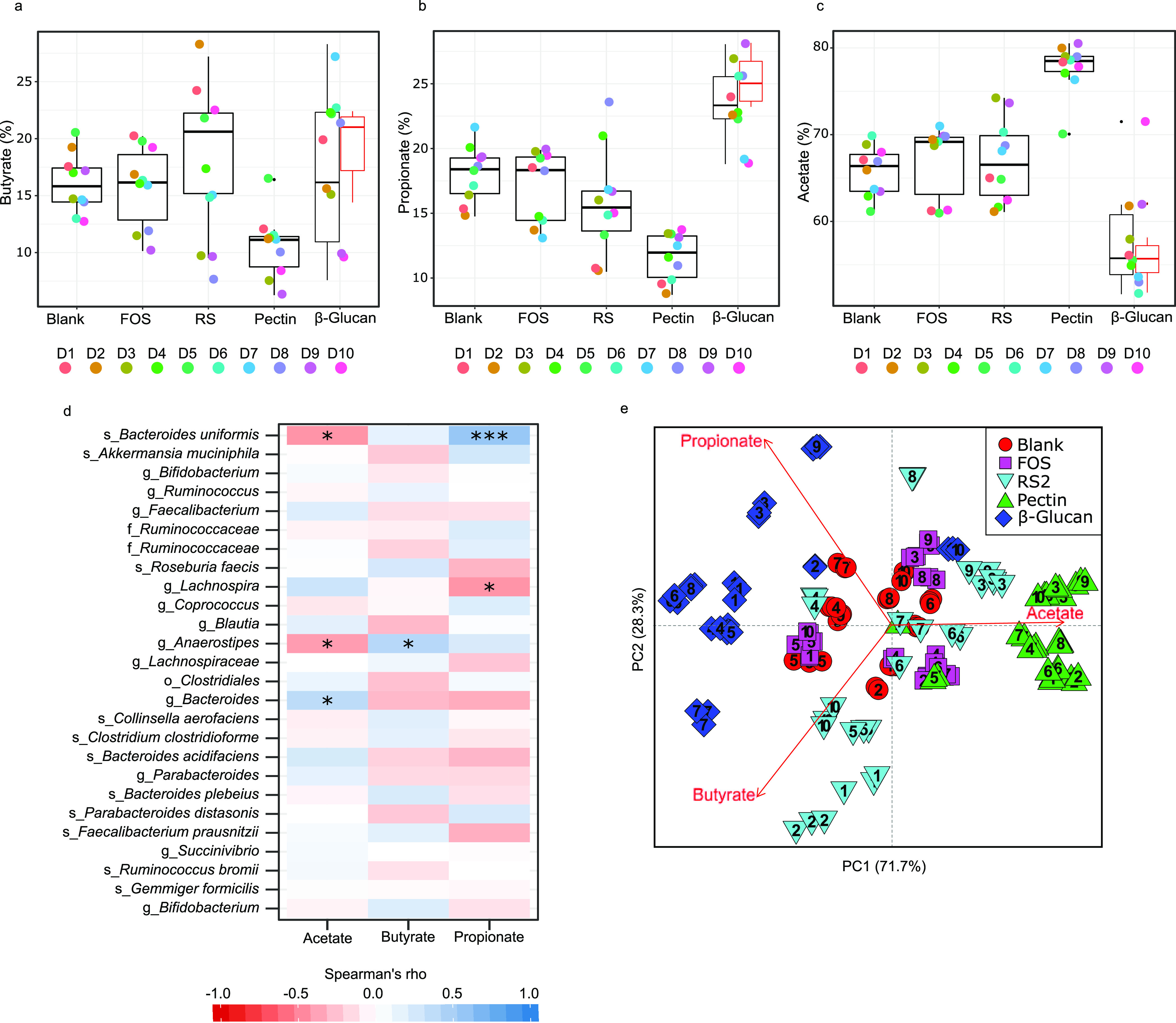
(a to c) SCFA percentage means after 24-h fermentation in each individual per substrate: butyrate (a), propionate (b), and acetate (c). (d) Spearman’s correlations (with FDR correction) in bacterial composition and relative proportions of SCFAs from the four interventions and the blank analyzed together. ASVs were collapsed at the species level and reported as the last classified taxonomic level (o, order; f, family; g, genus; s, species). The asterisks denote significance (*, *P* < 0.05; **, *P* < 0.01; ***, *P* < 0.001). (e) PCoA plot of butyrate, propionate, and acetate percentage means after 24-h fermentation. Numbers inside shapes indicate donors (D1 to D10).

Analysis of relative proportions of short-chain fatty acids (SCFAs) produced during the 24-h *in vitro* fermentation (butyrate, acetate, and propionate as a percentage of total SCFAs at 24 h of fermentation) and their associations with the relative abundance of specific bacteria assessed through Spearman’s correlation are shown in Fig. 6a to d. For the highly specific β-glucan, all donors had butyrate levels equal to or higher than 15% after fermentation, except for donors D9 and D10 (Fig. 6a). Furthermore, correlation analysis (Fig. 6d) showed that butyrate levels are associated with the abundance of unclassified *Anaerostipes* (*r_s_* = 0.41, *P* = 0.023), which was not present in D9 and D10. The overall consistency in high butyrate across donors fermented with the high-specificity fiber harboring unclassified *Anaerostipes* contrasts with the more individualized responses observed with samples of lower specificity such as RS2, which led to butyrate proportions as low as 7% or as high as 27%, depending on the donor (Fig. 6a). Propionate was also high in all donors fermented with the high-specificity β-glucan but was highly variable in other fiber ferments (Fig. 6b) and was positively correlated with B. uniformis (*r_s_* = 0.56, *P* < 0.001) (Fig. 6d). Unclassified *Anaerostipes* and B. uniformis were negatively associated with acetate production (*r_s_* = −0.39 and −0.43, *P* = 0.027 and 0.013, respectively), which is explained by the proportional increase of butyrate and propionate produced by these bacteria, respectively (Fig. 6d). For the intermediate-specificity pectin fiber, acetate was consistently increased in all donors, while butyrate and propionate presented some variation depending on donor (Fig. 6a to c). A principal-coordinate analysis (PCoA) of SCFA proportions in fecal ferments after 24-h fermentation clearly shows a tendency toward grouping to the left side of the plot for the β-glucan (except for D9 and D10) and grouping toward the right side of the plot for the pectin. RS2 and FOS, however, remained mostly spread in the center of the graph, together with the blank. After restricting analysis to individuals harboring bacteria targeted with the high-specificity fiber (*Anaerostipes* and B. uniformis), a significantly lower within‐donor dispersion was observed for the β-glucan compared to FOS and RS2 (PERMDISP *q* = 0.01 and *q* = 0.002, respectively) (see Table S1 in the supplemental material). Similarly, the intermediate-specificity pectin had lower within‐donor dispersion compared to FOS and RS2 (PERMDISP *q* = 0.02) and not different from that of the β-glucan (PERMDISP *q* = 0.692) (Table S1). Differences in centroid distances in PoCA plot, as evaluated by AMOVA, showed that significant changes (*P* value: <0.001) relative to the blank were promoted only by β-glucan and pectin but not by FOS or RS2 (Table S1). Thus, fibers of higher specificity resulted in more dramatic and similar shifts not only in gut microbiota structure across donors but also in metabolites produced, with more robust changes in SCFA profiles and higher interdonor similarity.

10.1128/mBio.01028-21.5 TABLE S1 Permutational analysis of multivariate dispersions (PERMDISP) and analysis of molecular variance (AMOVA) for short-chain fatty acids in pairwise comparisons among substrates. Download TABLE S1, DOCX file, 0.1 MB. Copyright © 2021 Cantu-Jungles et al. 2021 Cantu-Jungles et al. https://creativecommons.org/licenses/by/4.0/ This content is distributed under the terms of the Creative Commons Attribution 4.0 International license.

## DISCUSSION

In this study, we used an *in vitro* fecal fermentation model with 10 donors having divergent gut microbiota communities to show that a remarkably similar and more robust response can be obtained in targeted gut bacteria using high-specificity fibers but not low-specificity ones (as defined by Cantu-Jungles and Hamaker) (8). A critical challenge of the field has been that outcomes to fiber fermentations can be different in individuals with distinctly different community structures due to shifts in competitive pressures for substrate acquisition (6, 7, 14, 15). Thus, it has been difficult to imagine how to promote target microbes in a predictable way. We recently hypothesized that not all dietary fibers produce microbial responses that rely on the background gut microbial community, and we proposed a new hierarchical classification to rank dietary fibers (from low to high specificity toward gut microbes) related to targeting and consistency of response (8).

Dietary fibers are carbohydrate polymers and oligomers made up of one or more types of sugar units (e.g., xylose, fructose, glucose, galactose, arabinose, rhamnose, etc.) in 5- or 6-member rings of α or β anomericity which are bound together through glycosidic linkages that occur at different positions. Additionally, polymers of carbohydrate fibers can be arranged together resulting in physically complex networks with distinct three-dimensional arrangements that usually have poor solubility in water. Combinations of these features render dietary fibers with levels of physicochemical structures ranging from low complexity (e.g., those composed by a single sugar and linkage type, and soluble) to intermediate complexity (e.g., polymers of multiple sugar and linkage types, soluble) to high complexity (e.g., polymers of one or more sugar and linkage types networked into insoluble matrices). We proposed that high physicochemical complexity of dietary fibers limits the number of gut bacteria able to utilize them and thus increases fiber specificity. Moreover, from an evolutionary perspective, few microbes would have the genetic machinery to degrade fibers less commonly found in diets (8, 16), and fibers less utilized through cross-feeding mechanisms would also benefit a more limited number of colonic bacteria and thus present increased specificity (8). Highly specific fibers, i.e., fibers that alleviate competitive pressures for substrates due to their utilization by a narrow group of gut bacteria, would be able to promote a target bacterium regardless of the composition of the broader microbial community, relying only on the presence of the target microbe (8).

In this study, we showed that, in fact, fermentable fibers of high specificity to the target bacteria can promote their growth in different communities. The β-glucan of high specificity to unclassified *Anaerostipes* and B. uniformis, which we had identified previously (10), had a higher tendency toward clustering in a beta diversity plot among different donors than did less-specific fibers (FOS ≈ RS2 < pectin < β-glucan). Increased relative abundance of these two microbes was found in all donors who had any level of detectable sequences at the baseline. Although it is possible that there were undetectable sequences of these bacteria in nonresponders (D9 and D10 for unclassified *Anaerostipes* and D7 for B. uniformis), it is unlikely as they were not observed in any of the fiber ferments, and enrichment of unclassified *Anaerostipes* with β-glucan fermentation was observed even when baseline abundance was as low as 0.08%. The high-specificity fiber also resulted in more similar SCFA ratios across subjects with butyrate correlated with the abundance of unclassified *Anaerostipes* and propionate correlated with the abundance of B. uniformis. Thus, the highly specific β-glucan fiber was able to promote unclassified *Anaerostipes* and B. uniformis growth and metabolism independent of other bacterial members that were present in the community. We speculate that in contrast to high-specificity fibers, low-specificity ones were utilized by more gut microbes and thus were not causing significant shifts *in vitro* in any specific bacteria or the convergence of the overall community structure. Fibers of higher specificity promoted more dramatic shifts in microbial communities compared to baseline for both targeted microbes and beta diversity. Thus, reduction in competitive pressures not only means less competition and similarity in responses across individuals’ communities but also that more substrate will be available to fewer target bacteria that can grow efficiently and markedly change the gut microbial structure. This is in agreement with our previous observation that the use of a comparatively more specific soluble xyloglucan (i.e., more complex than pectin and FOS due to higher chemical complexity and because soluble xyloglucans are uncommon in diet) generated larger *in vitro* microbial shifts than pectin and FOS (17).

Our results imply that high-specificity fibers generate more dramatic gut microbial shifts than low-specificity ones, which has potential implications not only for the microbial ecology itself but also for aspects of clinical relevance. While there is a lack of *in vitro* studies evaluating responses to dietary fibers using several different human gut inocula, our data are in agreement with previously published literature on FOS and RS2 supplementation *in vivo* that indicates high interindividual variability in response to the supplementation of these fibers (18–20). Although some human studies suggest specific responses to FOS and RS2 based on analysis of means, bacterial shifts and metabolite production were not very consistent across individuals included in the studies. For instance, enrichment of *Lactobacillus* spp. and *Bifidobacterium* spp. after 16 days of inulin/oligofructose supplementation was different among subjects, and a portion of the individuals had a decrease in these bacteria, as indicated by quantitative PCR (20). The same study also showed a lack of hierarchical clustering of the HITChip data of individuals under the same treatment, also reflecting substantive interindividual variability. Similarly, another study using RS2 supplementation showed that it promoted increases in butyrate in only 22 out of 43 individuals, and shifts in important RS2 degraders like Bifidobacterium adolescentis and Ruminococcus bromii presented different trends in relative abundance (18). Interestingly, although we have initially placed RS2 as a low-to-intermediate-specificity fiber, it presented a low-specificity degree similar to that of FOS, with a high interindividual variability in microbiota response across donors, indicating that the transient physical limitation (insoluble form) at the beginning of fermentation is not as important to limit the number of bacteria able to benefit from subsequent resistant starch fermentation. Deehan et al. (3) showed in a human study that supplementation of chemically modified resistant starches (type 4 RS [RS4]), while presenting some degree of interindividual variability, still resulted in some consistent increments of specific bacteria across individuals. It seems probable that the RS4s were more specific than RS2 due to the chemical modifications affecting physical structure. Thus, the same class of polymer, depending on its structural characteristics, can potentially be placed in a different position in our hierarchical model of dietary fiber classification regarding specificity. Finally, target bacteria may not be present in all communities (as shown by the absence of unclassified *Anaerostipes* in D9 and D10 and absence of B. uniformis in D7), but theoretically such bacteria could be added to the community together with the high-specificity fiber in a synergistic symbiotic approach (i.e., aligned prebiotic + probiotic).

The study results point to a potential problem in comparing literature on dietary fiber and gut microbiota response. Differences in fiber responses across distinct microbial communities affect consistency among studies and could have the effect of minimizing the perceived potential of dietary fibers to modulate gut microbes (21). The extent to which the use of low- versus high-specificity dietary fibers influences the predictability of health outcomes is a matter of future clinical investigation. Moreover, some reports propose that individuals may have resilience to gut microbial shifts (i.e., nonresponders) with dietary manipulation (14, 15) but may be using low-specificity fibers. It has also been suggested that personalized approaches based on initial characteristics of the gut microbial community would be needed to effectively modulate gut microbial communities through diet (15, 19, 22). However, in the face of the complexity of the gut microbial community, prediction of outcomes from a given fiber utilized by many microbes relies on a sophisticated comprehension of all competitive interactions that occur in the gut (6). Although factors such as genetics and other environmental factors could also play a role in determining microbial responses to fibers *in vivo*, data show that available nutrients largely determine whether or not an organism can establish in a community (23–25). Thus, the “simplification” of this context presented here is to focus on reducing competitiveness for the substrate through the use of high-specificity dietary fibers and suggests a way forward to the effective modulation of distinct gut bacterial communities in a targeted and predictable way.

This proof-of-concept study has potential limitations. Four dietary fibers that we tentatively classified as low-, low-to-intermediate-, intermediate-, and high-specificity fibers were fermented *in vitro* by 10 different human gut microbial communities. We assume, however, that many other intermediate-specificity states occur, which were not evaluated here. Moreover, while parameters for fiber specificity classification are assumed to be chemical and physical complexity, commonality in the diet, and utilization through cross-feeding, a precise method to determine the degree of fiber specificity needs to be developed (8). This might include machine learning to link fiber structure and bacterial gene expression for fiber hierarchical classification. Moreover, while *in vitro* models are efficient to evaluate community effects on fiber utilization, they lack factors which could potentially affect outcomes *in vivo* such as the presence of other energy sources for bacteria (other dietary fibers from the diet and host mucins), bidirectional interactions of the host with the gut microbiota, and other environmental factors (26, 27). Thus, for a translational application, the specificity effects observed *in vitro* should be further confirmed *in vivo* in the human.

Overall, our *in vitro* results suggest that a strong growth can be promoted in target microbes embedded in any microbial community through the use of aligned high-specificity fibers, while outcomes from low-specificity fibers are more dependent on an individual’s microbial community structure and are often less dramatic and more individualized. Our data provide a new approach to design prebiotic or synergistic symbiotic-based interventions from the perspective of a hierarchical classification of dietary fibers based on specificity with the opportunity to be effective in a large number of subjects.

## MATERIALS AND METHODS

### Dietary fibers.

According to our proposed model (8), dietary fibers ranging from low to high specificity were selected based on features that could increase or decrease specificity, as described below.

### Low specificity.

Since fructooligosaccharides (FOSs) have a simple chemical structure (primarily β-2→1-linked fructans) and are commonly found in diets (28), it is expected that many microbes have evolved to utilize them and have the ability to degrade this simple linkage type. Moreover, the soluble nature of FOS allows easy microbial accessibility for its degradation. Previous studies indicate that many different gut microbes can degrade and utilize FOS for growth (29, 30) and corroborate the assumption that FOS has low specificity toward gut microbes. FOS from chicory (≥90% purity with a polymerization degree of 2 to 60 with an average degree of polymerization of >10) was obtained from Sigma (FOS; CAS number F8052).

### Low-to-intermediate specificity.

Type 2 resistant starch (RS2) also has a fairly simple chemical structure (α-1→4- and α-1→6-linked glucan) and is common in diets representing a large amount of insoluble fermentable dietary fiber arriving in the large intestine (31). It has an insoluble crystalline physical structure which is broken down by primary degraders to simple soluble oligomers and monomers that are released in the gut milieu to support a range of different bacteria (9, 18). Thus, RS2 was classified as a low-to-intermediate-specificity dietary fiber. Raw potato starch was obtained from Bob’s Red Mill (Clackamas, OR) and submitted to an *in vitro* upper gastrointestinal (GI) digestion procedure as previously described (32–34) to obtain the starch portion that is resistant to hydrolysis (RS2).

### Intermediate specificity.

Pectins are heteropolysaccharides which can harbor a high chemical complexity, comprising up to 17 different sugar units and containing more than 20 different linkages (35). On the other hand, pectins are soluble in water, which allows easy access of most gut microbes, and are present in the majority of plant food sources common in diets (35). Taking these factors together, we classified pectin as an intermediate-specificity dietary fiber. Pectin from citrus peel with galacturonic acid content of ≥74.0% with ≥6.7% methoxy groups (CAS number 9000-69-5) was obtained from Sigma-Aldrich (St. Louis, MO).

### High specificity.

Insoluble β-(1→3),(1→6)-glucan, a polymer, is composed of β-1→3 and β-1→6 linkages between glucose units. Insoluble β-(1→3),(1→6)-glucans are restricted to few food sources such as fungi and some yeast (36). Since this glucan is not as common in dietary patterns as the other fibers, we expected that fewer gut bacteria have evolved to contain the genetic machinery to hydrolyze and utilize it. More importantly, due to its insoluble nature, even bacteria that have the ability to degrade 1→3- and 1→6-linked glucans might not be able to access this polymer. In a previous study using a mixed fecal community, such a glucan from the mushroom Cookeina speciosa was shown to strongly promote a limited number of bacteria, namely, *Anaerostipes* and B. uniformis (10). Taken together, we supposed that few gut bacteria can access and utilize this β-glucan, and we classified it as a high-specificity dietary fiber. The insoluble β-glucan was isolated as described in the work of Cantu-Jungles et al. (10).

### Donors and fecal sample collection.

Fecal samples were obtained from 10 reportedly healthy donors who were consuming their routine diets and had not taken antibiotics for the last 6 months. Seven of the donors were male and three of them female in the age range of 26 to 42 years old (average, 35.1 years). All donors were within the normal body mass index (BMI) range (18.5 to 25 kg/m^2^). Fecal samples were collected in sterile plastic tubes which were immediately sealed, placed on ice, and then transferred into an anaerobic chamber (BactronEZ Anaerobic Chamber; Shel Lab, Cornelius, OR) where *in vitro* fecal fermentation was performed. All samples were utilized for fermentation within 1 h of collection. Human stool collection and use were approved by the Institutional Review Board at Purdue University (IRB protocol no. 1510016635).

### *In vitro* fecal fermentation procedures.

*In vitro* fermentations of the single dietary fibers (FOS, RS2, pectin, and β-glucan) and the blank (no fiber added) were performed in triplicate according to the methodology described in the work of Cantu-Jungles et al. (10) for the fecal samples from each of the 10 donors individually. Briefly, carbonate-phosphate buffer was prepared and sterilized by autoclaving at 121°C for 20 min. The buffer was then cooled to room temperature, oxygen was removed by bubbling with carbon dioxide, and cysteine hydrochloride (0.25 g/liter of buffer) was added as a reducing agent. The prepared buffer was then placed into the anaerobic chamber the day before experimentation to complete buffer reduction. On the day of experiment, the freshly collected fecal sample was homogenized with carbonate-phosphate buffer (1:3 [wt/vol]), followed by filtration through four layers of cheesecloth. Then, 1 ml of this fecal inoculum was added to Balch tubes (Chemglass Life Sciences, Vineland, NJ) containing 50 mg of the dietary fiber substrate and 4 ml of the carbonate-phosphate buffer. Tubes were closed with butyl rubber stoppers (Chemglass Life Sciences), sealed with aluminum seals (Chemglass Life Sciences), and incubated at 37°C in a shaker incubator (150 rpm; MaxQ 6000; Thermo Fisher, Waltham, MA) for 24 h. Aliquots of the baseline sample and samples after 24-h fermentation were prepared and stored at −80°C until further use for SCFA analysis (0.5 ml) and DNA sequencing (1 ml). All sample manipulation was conducted under an anaerobic atmosphere (85% N_2_, 5% CO_2_, and 10% H_2_).

### SCFA analysis.

Samples for SCFA analyses were prepared as previously described (10) and analyzed using a gas chromatograph (GC-FID 7890 A; Agilent Technologies Inc.) on a fused silica capillary column (Nukon Supelco no. 40369-03A; Bellefonte, PA) under the following conditions: injector temperature at 230°C, initial oven temperature at 100°C, and temperature increase of 8°C/min to 200°C with a hold for 3 min at final temperature. Helium was used as a carrier gas at 0.75 ml/min.

Quantification was performed based on relative peak area using external standards of acetate (A38S), propionate (A258), and butyrate (AC108111000) and an internal standard of 4-methylvaleric acid (AAA1540506) from Fisher Scientific (Hampton, NH).

### DNA extraction and 16S rRNA gene amplicon sequencing.

Stored samples for DNA extraction were thawed and centrifuged (13,000 rpm for 15 min), and supernatants were discarded. Automated DNA extraction of the precipitates was performed using the QIAcube Connect instrument (Qiagen, Germantown, MD) with the QIAamp PowerFecal Pro DNA kit (Qiagen) per manufacturer’s instructions. The V4 region of the 16S rRNA gene was amplified using primers 515F (5′-GTGCCAGCMGCCGCGGTAA) and 806R (5′-GGACTACHVHHHTWTCTAAT). The primers contained 5′ common sequence tags (known as common sequence 1 and 2 [CS1 and CS2]). First-stage PCR amplifications were performed in 10-μl reaction mixtures in 96-well plates, using MyTaq HS 2× master mix (Bioline, Memphis, TN). PCR conditions were 95°C for 5 min, followed by 28 cycles of 95°C for 30 s, 55°C for 45 s, and 72°C for 60 s. Amplicons were generated using a two-stage PCR amplification protocol as described previously (37). The primers contained 5′ common sequence tags (known as common sequence 1 and 2 [CS1 and CS2]) as described previously (38). Subsequently, a second PCR amplification was performed in 10-μl reaction mixtures in 96-well plates. A master mix for the entire plate was made using MyTaq HS 2× master mix. Each well received a separate primer pair with a unique 10-base barcode, obtained from the Access Array Barcode Library for Illumina (Fluidigm, South San Francisco, CA; catalog no. 100-4876). These Access Array primers contained the CS1 and CS2 linkers at the 3′ ends of the oligonucleotides. Cycling conditions were 95°C for 5 min, followed by 8 cycles of 95°C for 30 s, 60°C for 30 s, and 72°C for 30 s. Samples were then pooled in equal volume using an EpMotion5075 liquid handling robot (Eppendorf, Hamburg, Germany). The pooled library was purified using an AMPure XP cleanup protocol (0.6×, vol/vol; Agencourt, Beckman-Coulter, Indianapolis, IN) to remove fragments smaller than 300 bp. The pooled libraries, with a 20% phiX spike-in, were loaded onto an Illumina MiniSeq midoutput flow cell (2 × 153 paired-end reads). Based on the distribution of reads per barcode, the amplicons (before purification) were repooled to generate a more balanced distribution of reads. The repooled library was purified using AMPure XP cleanup, as described above. The repooled libraries, with a 20% phiX spike-in, were loaded onto a MiniSeq flow cell and sequenced (2 × 153 paired-end reads). Fluidigm sequencing primers, targeting the CS1 and CS2 linker regions, were used to initiate sequencing. Demultiplexing of reads was performed on instrument. Library preparation, pooling, and sequencing were performed at the University of Illinois at Chicago Genome Research Core (GRC) within the Research Resources Center (RRC).

### Bioinformatics.

Demultiplexed and preprocessed sequence reads were supplied as paired-end FASTQ sequence files and imported into QIIME 2 (q2) version 2019-10 for analysis (39). Amplicon sequence variants (ASVs) were generated using Deblur (40) (sequences were trimmed at 153 bp), and taxonomic assignment was carried out using the q2-*feature-classifier* plugin against the Greengenes reference database classifier with 99% similarity, specific for the V4 16S region (v. 3.8). To minimize the effects of sequencing depth on alpha and beta diversity measurement, the number of reads from each sample was rarefied to 3,600, which captured most of the sample’s richness and evenness as indicated by the Shannon index rarefaction curve (see Fig. S1 in the supplemental material). To understand interindividual differences of microbiota from donors included in the study, alpha and beta diversities were calculated using the q2-*diversity* plugin which included observed amplicon sequence variants (ASVs) and Pielou’s evenness index for alpha diversity and weighted and unweighted UniFrac distances for beta diversity.

10.1128/mBio.01028-21.1 FIG S1 Alpha diversity metrics as determined by Shannon index of samples pooled by treatment type as a function of sampling depth with no subsampling applied. Download FIG S1, EPS file, 0.1 MB. Copyright © 2021 Cantu-Jungles et al. 2021 Cantu-Jungles et al. https://creativecommons.org/licenses/by/4.0/ This content is distributed under the terms of the Creative Commons Attribution 4.0 International license.

Sequence alignment and construction of a phylogeny tree were obtained using the Qiime2 pipeline *align-to-tree-mafft-fasttree*. Statistical differences in alpha diversity were calculated using q2-*alpha-group-significance* plugin. For beta diversity, baseline microbial communities were compared through permutational multivariate analysis of variance (PERMANOVA) (41). To evaluate within-treatment similarity of microbial communities from the different donors, dispersions of donors in weighted UniFrac diversity plot under the same fiber fermentation were compared to the blank through homogeneity of multivariate dispersions (PERMDISP) (42). To determine whether centroid locations after fermentation of substrates were significantly different, weighted UniFrac distance matrices were uploaded into mothur (v. 1.37.6) and evaluated through analysis of molecular variance (AMOVA) (43) using the amova function. Pairwise distances (changes in weighted UniFrac) and differences (changes in PC1 and PC2 from the weighted UniFrac plot) before and after fiber fermentation were analyzed through Wilcoxon signed-rank test followed by Kruskal-Wallis tests to evaluate between-treatment significance as implemented in the q2-*longitudinal* plugin (44). Taxon bar plots of relative abundance at baseline were generated with the q2-*taxa* plugin with ASVs collapsed at the phylum level. The ASV table was generated by collapsing nonrarefied ASVs at the species level for the different substrate ferments and was further used to calculate relative abundances. The ASV table was also used for detection of differentially abundant taxa across fiber substrates using a linear mixed-effects model implemented in ANCOM-II (Analysis of Composition of Microbiomes-II) R-code (https://github.com/FrederickHuangLin/ANCOM), accounting for the donor as the random effect and adjusting for time (before versus after substrate fermentation). Each fiber treatment was run separately against the blank in ANCOM-II, and a taxon was determined to be significant if it passed the 0.9 cutoff. Relative abundance of species before and after fiber fermentation, plots of relative proportion of each SCFA produced, Spearman’s correlation analysis (with FDR correction) between bacterial composition (with a mean relative abundance of >1%) and relative proportions of SCFAs, and principal-coordinate analysis (PCoA) of SCFAs were performed in R Stats software version 3.5.1 (R Core Team, Vienna, Austria). The SCFA distance matrix generated for the PCoA plot was also loaded into mothur and Qiime2 for AMOVA and PERMDISP analyses, respectively.

### Data availability.

Raw reads are available in the National Center for Biotechnology Information Sequence Read Archive (NCBI; SRA), BioProject PRJNA640404, and BioSamples SAMN15317177 to SAMN15317354. All code is freely available at the GitHub repository (https://github.com/ThaisaJungles/fiber_specificity).
