# Supplementary material for: Dietary Fiber Hierarchical Specificity: the Missing Link for Predictable and Strong Shifts in Gut Bacterial Communities
Source: mBio. 2021 Jun 29;12(3):e01028-21. doi: 10.1128/mBio.01028-21 (PMC8262931; doi:10.1128/mBio.01028-21)
Supplement: TABLE S1 [file mbio.01028-21-st001.docx]

Table S1. Permutational analysis of multivariate dispersions (PERMDISP) and analysis of molecular variance (AMOVA) for short chain fatty acids in pairwise comparisons among substrates

| **Substrate** | | **Sample size** |  | **PERMDISP** | | |  | **AMOVA** | |
| --- | --- | --- | --- | --- | --- | --- | --- | --- | --- |
|  |  |  |  | **F-value** | **p-value** | **q-value** |  | **Fs** | **p-value** |
| Blank | FOS | 59 |  | 8.437956 | 0.004 | 0.006667 |  | 1.55733 | 0.204 |
|  | Glucan | 49 |  | 0.146199 | 0.692 | 0.692 |  | 98.4013 | <0.001 |
|  | Pectin | 59 |  | 1.019949 | 0.14 | 0.175 |  | 169.709 | <0.001 |
|  | RS | 59 |  | 36.27278 | 0.001 | 0.002 |  | 2.37941 | 0.116 |
| FOS | Glucan | 50 |  | 6.029471 | 0.007 | 0.01 |  | 82.7673 | <0.001 |
|  | Pectin | 60 |  | 10.30054 | 0.001 | 0.002 |  | 93.82 | <0.001 |
|  | RS | 60 |  | 12.24405 | 0.001 | 0.002 |  | 0.837544 | 0.414 |
| Glucan | Pectin | 50 |  | 0.211839 | 0.637 | 0.692 |  | 460.299 | <0.001 |
|  | RS | 50 |  | 24.11772 | 0.001 | 0.002 |  | 43.7505 | <0.001 |
| Pectin | RS | 60 |  | 36.3865 | 0.001 | 0.002 |  | 51.1896 | <0.001 |

(P-value is based on 999 permutations for PERMDISP and 1000 permutations for AMOVA)
